# Optimization Model of Signal-to-Noise Ratio for a Typical Polarization Multispectral Imaging Remote Sensor

**DOI:** 10.3390/s22176624

**Published:** 2022-09-01

**Authors:** Ying Zhang, Hao Wang, Heshen Li, Junhua Sun, Huilan Liu, Yingshuo Yin

**Affiliations:** School of Instrumentation Science & Opto-Electronics Engineering, Beihang University, No. 37 Xueyuan Road, Haidian District, Beijing 100191, China

**Keywords:** signal-to-noise ratio, polarization, spectral, remote sensor, 6SV–SNR coupling model

## Abstract

The signal-to-noise ratio (SNR) is an important performance evaluation index of polarization spectral imaging remote sensors. The SNR-estimation method based on the existing remote sensor is not perfect. To improve the SNR of this model, a partial detector check slant direction is presented in this study, and a polarization extinction ratio related to the internal SNR model of a typical multispectral imaging remote sensor is combined with the vector radiative transfer model to construct the atmosphere 6SV–SNR coupling model. The new result is that the central wavelength of the detection spectrum, the observation zenith angle, and the extinction ratio all affect the SNR of the remote sensor, and the SNR increases with the increase in the central wavelength of the detection spectrum. It is proved that the model can comprehensively estimate the SNR of a typical polarization multispectral imaging remote sensor under different detection conditions, and it provides an important basis for the application evaluation of such remote sensors.

## 1. Introduction

In recent years, with the development of optical technology and the increase in the demand for optical remote sensing information in various fields, polarization multispectral imaging technology has become a hot research topic in the field of aviation and ground remote sensing technology [1,2,3,4,5,6]. Based on the rotating polaroid and polarization filter multispectral imaging remote sensor, polarization multispectral imaging can be realized to detect the most typical type of remote sensor. The SNR of a remote sensor is an important factor for determining whether a target can be detected or not, and an important index for measuring the imaging quality and radiation performance of the remote sensor [7,8,9,10,11,12,13].

In 1993, Bo-Cai in the United States used the local mean and local standard deviation of small image blocks to calculate the average SNR of unsupervised images, where the noise is additive [14]. However, this method is not suitable for targets with small spatial scales because it does not consider the interband effect. In 1998, Curtis Earl Volin reported results from a simple SNR analysis of the computed tomography imaging spectrometer (CTIS) and pointed out that the SNR of the CTIS is proportional to the square root of the dwell time [15]. In 2001, JTL Thong and KS Sim proposed a method to estimate the SNR from a single image. The method uses autocorrelation techniques and assumes that noise is irrelevant from one pixel to another. The experimental results show that the method has a good approximation in SEM images [16]. In 2005, R. Glenn Sellar and Glenn D. Boreman compared the SNR of different types of imaging spectral remote sensors and proposed relative SNR coefficients to laterally compare the SNR of various types of imaging spectral remote sensors [8]. In 2013, Wang took into account the quantification of the atmospheric scattering effect in view of the traditional SNR model, which mainly focused on optical parameters and detector characteristics. The results show that scattered light accounts for a large part of the remote sensor’s dynamic range, which leads to significant SNR attenuation [17]. In 2018, Su from Changguang Satellite Technology Co., Ltd., studied the SNR of the CMOS imaging remote sensor, proposed an SNR model based on digital domain TDI technology, and verified the accuracy of the model through a radiation calibration experiment using a space optical camera [18]. A.R. Chen captured an optical image with one backplane IC implemented in standard 0.18 micro-m CMOS technology incorporating display drivers and an array of avalanche diodes [19]. In 2021, Wang and Zhao developed a formula for calculating the SNR by summarizing the model of the imaging remote sensor and concluded that the image SNR decreases with an increase in the number of luminous points of the target when the other conditions are the same [20].

The research on the SNR of instruments is developing from simple remote sensors to the full-chain type. For the polarization spectral imaging remote sensor, considering the polarization characteristics, it is necessary to establish an SNR-estimation model suitable for the polarization spectral imaging remote sensor, which is indispensable to the study of the atmospheric radiation transmission process.

At present, the research on the SNR is mainly focused on the interior of the sensors and denoising methods [21,22,23,24], and mostly the SNR of the spectral remote sensors, rather than the SNR of the remote sensors combined with polarization, has been deeply studied. However, the estimation methods of the SNR of polarized multispectral imaging remote sensors combined with the atmospheric radiative transfer process are not perfect. This paper combines the SNR-calculation formula of the traditional imaging remote sensor with the structural parameters of the typical polarization multispectral imaging remote sensor based on a rotating polarizer. In addition, the internal SNR model of a typical polarization multispectral imaging remote sensor is established, which is related to the detection direction and extinction ratio of the polarizer. Combined with the atmospheric vector radiative transfer model, a 6SV–SNR coupling model based on the imaging chain of the solar atmosphere, air target, target remote sensor, and internal imaging of the remote sensor was constructed. The SNR variation of a typical polarization multispectral imaging remote sensor under different detection conditions is analyzed through the control parameter input to provide a reference for selecting a suitable polarization multispectral imaging technology.

## 2. Methods

The 6SV–SNR coupling model is built for a typical polarization multispectral imaging remote sensor, therefore the polarization detection principle and noise analysis should be presented first, as shown in Section 2.1 and Section 2.2. On this basis, the analysis and derivation process of the internal SNR model of the remote sensor and the 6SV–SNR coupling model are presented in Section 2.3.

### 2.1. Polarization Detection Principle

The asymmetry of the amplitude or vibration direction of light with respect to the direction of propagation is called the polarization of light [25]. Various states of polarized light can be described by the ellipse of polarization, but it is only applicable to describe completely polarized light, and our lives include unpolarized light and partially polarized light. To eliminate the limitation of the description of the ellipse of polarization, in 1852, George Gabriel Stokes proposed the Stokes vector method to describe all forms of light in nature. The advantage of the Stokes vector method is that all parameters in the Stokes vector can be measured directly. The Stokes vector contains four parameters, which are expressed as [26,27]:

(1) $$S=\left[\begin{array}{l} S_{0}\\ S_{1}\\ S_{2}\\ S_{3} \end{array}\right]=\left[\begin{array}{l} I\\ Q\\ U\\ V \end{array}\right]=\left[\begin{array}{l} I_{0^{\circ }}+I_{90^{\circ }}\\ I_{0^{\circ }}-I_{90^{\circ }}\\ I_{45^{\circ }}-I_{135^{\circ }}\\ I_{R}-I_{L} \end{array}\right]$$

where *I* represents the total light intensity; *Q* represents the light-intensity difference of linearly polarized light in the direction of 0° and 90°; *U* represents the light-intensity difference of linearly polarized light in the 45° and 135° directions; *V* represents the light-intensity difference between the right and left circularly polarized light components.

In 1943, the Mueller matrix was proposed to describe the change in the polarization state of light after passing through linearly polarized optics. The Mueller matrix is a 4 × 4 matrix with the form

(2) $$M=\left[\begin{array}{c} m_{00}\;m_{01}\;m_{02}\;m_{03}\\ m_{10}\;m_{11}\;m_{12}\;m_{13}\\ m_{20}\;m_{21}\;m_{22}\;m_{23}\\ m_{30}\;m_{31}\;m_{32}\;m_{33} \end{array}\right]$$

Assuming that the polarization state of the incident light is expressed as *S_i_*, and the polarization state of the outgoing light after passing through the polarization remote sensor is expressed as *S_o_* by the Stokes vector, the relationship between them can be described by the Mueller matrix as [26]

(3) $$S_{o}=M\times S_{i}$$

If there are multiple polarization elements in the remote sensor, then there will be multiple Mueller matrices (*M*1, *M*2, *M*3…). At this point, the relationship between the incident light *S_i_* and the outgoing light *S_o_* can be expressed as

(4) $$S_{o}=M_{n}\times \ldots M_{2}\times M_{1}\times S_{i}=M\times S_{i}$$

The light intensity detected by the remote sensor and the polarization state of the incident light can be expressed by the first line of the Mueller matrix. Monolithic integration on *S_i_* platform has emerged as an attractive approach to realize opto-electronic integration, as it is easily scaled to large arrays of devices in CMOS process [28], so that the light intensity could be detected by the remote sensor.

(5) $$I_{o}=\left[\begin{array}{c} m_{00}\;m_{01}\;m_{02}\;m_{03} \end{array}\right]\times S_{i}$$

Therefore, the polarization remote sensor can be used for four measurements to construct four sets of equations to obtain the polarization information of the incident light, namely

(6) $$\left[\begin{array}{c} I_{1}\\ I_{2}\\ I_{3}\\ I_{4} \end{array}\right]=\left[\begin{array}{c} m_{00}{\;m}_{01}{\;m}_{02}{\;m}_{03}\\ m_{10}{\;m}_{11}{\;m}_{12}{\;m}_{13}\\ m_{20}{\;m}_{21}{\;m}_{22}{\;m}_{23}\\ m_{30}{\;m}_{31}{\;m}_{32}{\;m}_{33} \end{array}\right]\times \left[\begin{array}{c} I\\ Q\\ U\\ V \end{array}\right]=M\times \left[\begin{array}{c} I\\ Q\\ U\\ V \end{array}\right]$$

where, *I*_1_, *I*_2_, *I*_3_, and *I*_4_ represent the four light-intensity values detected by the remote sensor respectively. To ensure that the data of the four measurements are linearly independent, and that *M* is reversible, then

(7) $$\left[\begin{array}{c} I\\ Q\\ U\\ V \end{array}\right]=M^{-1}\times \left[\begin{array}{c} I_{1}\\ I_{2}\\ I_{3}\\ I_{4} \end{array}\right]$$

According to (7), the Stokes vector parameters of incident light can be obtained. In practical polarization detection, the circular polarization component has little influence; thus, the circular polarization component is ignored, and the linear polarization characteristic is mainly studied.

### 2.2. Noise Analysis

The polarization spectral imaging remote sensor belongs to the imaging apparatus; thus, the detection and imaging of the production of each link will likely have noise, including detector noise, photon noise, and quantization noise. The noise source for the imaging instruments is mainly from two parts: the imaging remote sensor itself with the noise of the detector and the target or background radiation signal, which generates the photon noise. Therefore, we analyzed the noise of the polarization spectral imaging remote sensor based on the detector noise and photon noise.

The detector noise is related to the sensor manufacturing process and is the most basic and inevitable noise of the imaging remote sensor, which mainly includes dark-current noise, readout noise, and transfer noise. In recent years, with the continuous improvement of sensor manufacturing technology, the transfer noise has become negligible, whereas the dark-current noise and readout noise have become the main types of detector noise.

The dark current noise (*N_dark_*) is generated by the thermal motion of electrons and holes. The size of the dark current noise is closely related to the ambient temperature of the material, and it is generated in different components and layers of the material, including the back dark current, basement dark current, diffuse dark current, and other aspects. The dark current represents the generation rate of thermoelectric charge, and the dark current noise generated on each pixel can be expressed as:

(8) $$Dark\left(e^{-}\right)=AT^{1.5}e^{-E/2kT}$$

where, $e^{-}$ is the electron (negatively charged, e is the charge); *A* is a constant whose value can be measured experimentally; *T* is the temperature of the material when the sensor is working; *E* is the bandgap energy of the material, and *k* is Boltzmann’s constant, The larger *A*, *T*, and *k* are, the greater *N_dark_* is, the greater *N_noise_* is; the smaller *E*, the smaller *N_dark_* is, the smaller *N_noise_* is.

The dark-current noise of the detector brings two types of noise to the remote sensor: the dark-scatter noise and the dark-current non-uniformity noise. The dark-scatter noise follows the Poisson distribution and describes a random process, whose magnitude can be calculated by the following formula:

(9) $$N_{darks}=\sqrt{Dark\left(e^{-}\right)}$$

The specific value of the dark-current non-uniformity noise can be calculated using the following formula:

(10) $$N_{darku}=\varepsilon \cdot Dark\left(e^{-}\right)$$

where *ε* is the non-uniformity coefficient of the dark current, which represents the difference in the dark current between different pixels. Therefore, the total dark-current noise can be calculated by

(11) $$N_{dark}\left(e^{-}\right)=\sqrt{Dark\left(e^{-}\right)+{\left(\varepsilon \cdot Dark\left(e^{-}\right)\right)}^{2}}$$

It can be noted from this formula that the dominant position of the dark-current scatter particle noise and dark-current non-uniformity noise will vary when the amount of dark-current charge is different. When the amount of charge is small, the dark-current noise is dominant. When the charge amount is large, the noise of the dark-current inhomogeneity is dominant.

The electronic readout noise (*N_read_*) is proportional to the sampling frequency and the frame rate of the visible light camera in the case of visible light cameras [29]. Therefore, doubling the sampling frequency will also double the value of the electronic readout noise, which can be used as a reference value in the device manufacturer’s instructions.

(12) $$N_{read}\propto \left(\omega ,\upsilon \right)$$

where ω is the sampling frequency and υ is the frame rate of the visible light camera.

The transfer noise represents a random process of charge loss and increase, and because it is a random process, it also appears in the form of noise. The main reason is the capture of the interface state and internal state when the charge is transferred between pixels. The value of transfer noise can be calculated by

(13) $$N_{CT}\left(e\right)=\sqrt{\frac{2\times N\times Signal(e)}{CTE}}$$

The photon noise (*N_light_*) is caused by fluctuations in the number of photons around a certain mean because a photon is a boson conforming to Bose–Einstein statistics. In a broad sense, photon noise includes quantum noise and wave noise. The former is the fluctuation caused by completely independent photon emission, and the latter reflects the correlation between photon emission and frequency. Photon noise refers to quantum noise, which conforms to the Poisson distribution and is also a random process [30], with an equal expected value and variance.

After sampling by the remote sensor, photon fluctuation changes to electron fluctuation, but it still obeys the Poisson distribution. The variance of photon noise is proportional to the number of electrons converted to reach the detector target surface, namely:

(14) $$\sigma_{p}^{2}\propto I\left(x_{n}\right)\;\mathrm{or}\;\sigma_{p}^{2}=\eta I\left(x_{n}\right)$$

Therefore, the influence of the dark-current noise, readout noise, and photon noise on the SNR of the remote sensor is mainly considered when the SNR model of the polarization spectral imaging remote sensor is established. Therefore, when establishing the SNR model, the total noise can be expressed as

(15) $$N_{noise}=\sqrt{N_{dark}^{2}+N_{read}^{2}+N_{light}^{2}}$$

where *N_dark_*, *N_read_*_,_ and *N_light_* represent the dark-current noise, readout noise, and photon noise, respectively. The dark-current noise and readout noise can be calculated from the reference values in the detector instruction manual. The photon noise *N_light_* = N_P_^1/2^, where N_P_ represents the total number of electrons reaching the detector target. *N_dark_*, *N_read_*_,_ and *N_light_* all affect the total noise (*N_noise_*): the bigger they are, the bigger *N_noise_* is, and according to (22), the SNR decreases as *N_noise_* increases.

### 2.3. SNR Model

#### 2.3.1. Internal SNR Model of Typical Polarization Multispectral Imaging Remote Sensor Based on Rotating Polarizer

**Figure 1:**
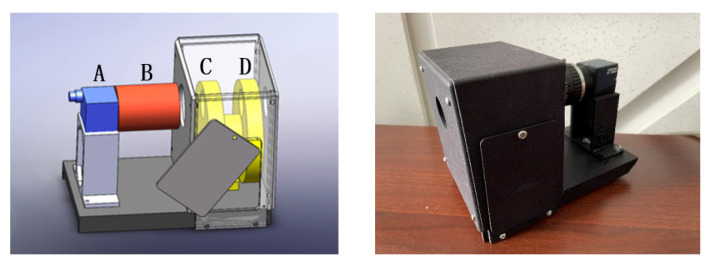
Super-resolution image reconstruction. (**Left**): remote sensor model diagram (A, camera; B, optical lens; C, filter; D, polarizer). (**Right**): physical remote sensor prototype.

This study selects the most classical polarization spectral imaging remote sensor, whose main components are the polarizer, filter, optical lens, and camera, as shown in Figure 1. This remote sensor can obtain the polarization characteristics of objects in the detection scene, can meet the needs of polarization spectral imaging detection, and has the advantages of simple operation and portability.

The polarizer changes the polarization state of light, and the energy through the polarizer varies in different polarization directions, that is, the polarization direction of the polarizer is closely related to the intensity, thus affecting the SNR of the remote sensor. The effect of the filter on the SNR mainly lies in its optical transmittance. The aperture of the lens affects the amount of light in the remote sensor and consequently the SNR. In addition, the theoretical value of lens transmittance in the visible range is approximately 92%, whereas the transmittance value is approximately 90% when material absorption is considered.

The SNR modeling of the remote sensor firstly considers the generation and transmission of signals, and we temporarily ignore the radiative transmission of signal energy in the atmosphere. We collectively refer to the incident light as the sunlight that reaches the pupil of the remote sensor through atmospheric radiation transmission and target reflection. In essence, the imaging mode of the polarization spectral imaging remote sensor is consistent with that of the traditional imaging remote sensor. Therefore, the traditional SNR calculation formula of the imaging remote sensor is applicable to the polarization spectral imaging remote sensor, and the signal-energy-calculation formula is expressed as:

(16) $$N_{e0}=L_{\lambda }\cdot \frac{\pi }{4F^{2}}\cdot \frac{\lambda }{hC}\cdot A_{d}\cdot \tau \cdot \eta \cdot \Delta \lambda \cdot T$$

where *L_λ_* queue is the radiance of the remote sensor’s entry pupil light; F is the F number of the remote sensor, also known as the aperture number, and in this remote sensor, it is the ratio of the focal length of the lens to the aperture diameter; *A_d_* is the area of the detector pixel; *λ* is the wavelength of the remote sensor; *τ* is the transmittance of the optical remote sensor; *η* is the quantum conversion efficiency of the detector; *h* is the Planck constant 6.624 × 10^−34^ J·S; *c* is the speed of light in vacuum and is approximately 3 × 10^8^ m/s; *T* is the integration time of image acquisition; Δ*λ* is the spectral bandwidth (half peak width) of the filter.

In the polarization spectral imaging remote sensor, the predecessors only obtained the light wave energy after passing through the optical device by using the radiance and optical transmittance. However, due to the existence of polarizers, the change of the polarization state of incident light when passing through the remote sensor needs to be taken into account., and the first term in the Stokes vector of incident light represents the total light intensity. Therefore, in this study, the SNR model of the polarization spectral imaging remote sensor is established using the light intensity instead of the entry pupil radiance. Assuming that the polarization state of the incoming pupil light is *S_in_* = [*I_in_ Q_in_ U_in_ V_in_*]^T^, after entering the polarization spectral imaging remote sensor, the Mueller matrix of the ideal polarizer is

(17) $$M_{0}=\frac{1}{2}\left[\begin{array}{cccc} 1 & \cos2\alpha & \sin2\alpha & 0\\ \cos2\alpha & \cos^{2}2\alpha & \cos2\alpha \sin2\alpha & 0\\ \sin2\alpha & \cos2\alpha \sin2\alpha & \sin^{2}2\alpha & 0\\ 0 & 0 & 0 & 0 \end{array}\right]$$

where *α* is the polarization angle of the polarizer. To improve the accuracy, this study considers the influence of the extinction ratio of the polarizer on the polarization information and optimizes the Mueller matrix above to obtain the Mueller matrix of the actual polarizer.

(18) $$\begin{array}{l} M_{new}\\ =\frac{\tau_{2}^{2}}{2}\left[\begin{array}{cccc} \varepsilon^{2}+1 & \left(\varepsilon^{2}-1\right)\cos2\alpha & \left(\varepsilon^{2}-1\right)\sin2\alpha & 0\\ \left(\varepsilon^{2}-1\right)\cos2\alpha & \left(\varepsilon^{2}+1\right)\cos^{2}2\alpha +2\varepsilon \sin^{2}2\alpha & {\left(\varepsilon -1\right)}^{2}\sin2\alpha \cos2\alpha & 0\\ \left(\varepsilon^{2}-1\right)\sin2\alpha & {\left(\varepsilon -1\right)}^{2}\sin2\alpha \cos2\alpha & \left(\varepsilon^{2}+1\right)\sin^{2}2\alpha +2\varepsilon \cos^{2}2\alpha & 0\\ 0 & 0 & 0 & 2\varepsilon \end{array}\right] \end{array}$$

where $\tau_{1}$ and $\tau_{2}$ are the maximum and minimum amplitude transmittance of the polarizer in a certain transmittance direction, respectively, and $\varepsilon^{2}={\left(\tau_{1}/\tau_{2}\right)}^{2}$ is the extinction ratio of the polarizer. Therefore, the polarization state *S_out_* of the incoming pupil light after passing through the polarizer can be expressed as:

(19) $$S_{out}=M_{new}\times S_{in}$$

Special attention should be paid to the change in the total-light intensity-value when establishing the SNR model. As can be observed from (5), the change in the total-light-intensity value after the pupil light passes through the polarizer is only related to the first row of the Mueller matrix of the polarizer, which can be expressed as:

(20) $$I_{out}=\frac{\tau_{2}{}^{2}}{2}\left[I_{in}(\varepsilon^{2}+1)+Q_{in}\left(\varepsilon^{2}-1\right)\cos2\alpha +U_{in}\left(\varepsilon^{2}-1\right)\sin2\alpha \right]$$

In photometry, light intensity is the luminous flux per unit solid angle, and brightness is the unit solid angle of the luminous flux per unit area. Thus, the spin polarization of the polaroid spectral imaging remote sensor is used to model the SNR. If the light intensity is greater than the radial brightness divided by the aperture area, then the improved signal-energy-computation formula is expressed as

(21) $$\begin{array}{l} N_{e}\\ =\frac{\tau_{2}{}^{2}}{2}\left[I_{in}(\varepsilon^{2}+1)+Q_{in}\left(\varepsilon^{2}-1\right)\cos2\alpha +U_{in}\left(\varepsilon^{2}-1\right)\sin2\alpha \right]\cdot \frac{1}{D^{2}F^{2}}\cdot \frac{\lambda }{hC}\cdot A_{d}\cdot \tau \cdot \eta \cdot \Delta \lambda \cdot T \end{array}$$

where *I_in_*, *Q_in_*, and *U_in_* are the first three components of the Stokes vector for the incoming pupil light, respectively. α is the transmissibility angle of the polarizer. After determining the Stokes vector of the incident light, we can use the signal-energy-calculation formula of the traditional imaging spectrometer to complete the signal-energy modeling of the polarization spectral imaging remote sensor based on the rotary polarizer in the direction of deviation α. Combined with the above noise model, the polarization spectral imaging remote sensor model based on a rotating polarizer is completed.

(22) $$\begin{array}{l} SNR_{\alpha }=\frac{N_{e}}{N_{noise}}\\ =\frac{\frac{\tau_{2}{}^{2}}{2}\left[I_{in}(\varepsilon^{2}+1)+Q_{in}\left(\varepsilon^{2}-1\right)\cos2\alpha +U_{in}\left(\varepsilon^{2}-1\right)\sin2\alpha \right]\cdot \frac{1}{D^{2}F^{2}}\cdot \frac{\lambda }{hC}\cdot A_{d}\cdot \tau \cdot \eta \cdot \Delta \lambda \cdot T}{\sqrt{N_{dark}^{2}+N_{read}^{2}+N_{light}^{2}}} \end{array}$$

#### 2.3.2. Proposed 6SV–SNR Coupling Model

The Laboratory of Atmospheric Optics at Lille University of Science and Technology in France developed 5S [31] (simulation of the satellite signal in the solar spectrum), which can simulate the process of atmospheric radiation transmission and calculate the brightness of the satellite’s entry pupil. In 1997, Eric Vemote added the latest scattering calculation method based on 5S, which improved the calculation accuracy and took into account the problems of molecular absorption, aerosol scattering, and heterogeneous ground. The second simulation of the satellite signal in the solar spectrum was proposed by shortening the step of spectral integration from the original 5 nm to 2.5 nm [32]. In 2006, Svetlana Y. Kotchenova and Eric F.Vermote introduced a vector model 6SV by considering polarization based on 6S [1]. The 6SV uses the continuous order scattering method, which can calculate the Stokes vector of the light coming in and out of the pupil, and the calculation accuracy is 0.4–0.6%. In this study, the 6SV calculation software was used to establish the atmospheric vector radiative transfer model, and the specific modeling process is composed of the following:

1. Weather conditions:

Based on the actual weather conditions when setting the atmospheric model, determine the atmospheric level, boundary height, atmospheric extinction, and scattering type; set the aerosol model; determine the composition of atmospheric molecules and their scattering and extinction coefficients; set the weather visibility parameters according to the actual situation.

2. Detection geometry:

Select the time and place of modeling (latitude and longitude). According to the time and place information, use computing software to calculate the solar zenith and azimuth angles, as the solar azimuth information input 6SV. The zenith and azimuth angles of the detector are input according to the actual detection requirements.

3. Detection parameters:

Select the band model. The purpose of this study is to investigate the atmospheric vector radiative transfer characteristics under multiple wavelengths. Choose the single-band pattern modeling, input the detection of the wavelength of each probe channel, the object detection for the uniform or non-uniform surface, as well as the target reflection type, single-band reflectance, and polarization parameters on the surface of the target. The polarization parameters of the target surface are the Q and U parameters of the light emitted from the object measured in the chamber at a specific observation angle.

For accurate modeling, the atmospheric model should take advantage of the high-precision detector used to retrieve the atmospheric parameters. A custom atmospheric profile was derived using the required parameters, such as temperature, water-vapor density, and ozone density; however, the actual measurement process is complicated and changes at any time owing to atmospheric conditions, making it unsuitable for high-precision measuring instruments. Therefore, the typical atmospheric model in 6SV was used in this study. Because 6SV was used as the forward modeling process in this study, the atmospheric correction was not considered.

**Figure 2:**
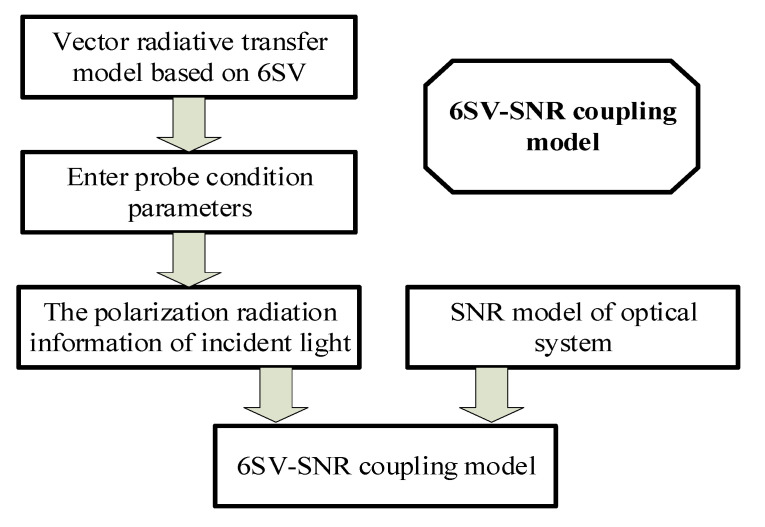
Coupling modeling process of the 6SV–SNR (Firstly, the parameters are input into the vector radiative transfer model based on 6SV to obtain the polarization radiation information of incident light. Secondly, the SNR model of optical remote sensor is established. Finally, the SNR of the optical remote sensor can be obtained by substituting the polarization radiation information of the incident light into the SNR model).

The atmospheric vector radiation transfer model based on 6SV can simulate the radiance of the solar radiation wave transmitted by the atmospheric vector to the pupil of the remote sensor, as well as obtain the polarization radiation information of the incident light. By coupling it with the SNR model, a full-chain SNR model of the polarization spectral remote sensor considering each link of the SNR imaging chain can be obtained. The 6SV–SNR coupling modeling process is shown in Figure 2.

Through the input of weather conditions, detection geometry conditions, and detection parameters, the polarization radiation information $\vec{L_{s}}$ of the incident light at the pupil of the remote sensor was obtained using the atmospheric vector radiative transfer model based on 6SV. Combined with the internal SNR model of the remote sensor, the 6SV–SNR coupling model can be expressed as

(23) $$SNR_{\alpha }=\frac{N_{e}}{N_{noise}}=\frac{M_{nr1}\cdot \vec{L_{s}}\cdot \frac{1}{D^{2}F^{2}}\cdot \frac{\lambda }{hC}\cdot A_{d}\cdot \tau \cdot \eta \cdot \Delta \lambda \cdot T}{\sqrt{N_{dark}^{2}+N_{read}^{2}+N_{light}^{2}}}$$

where *M*_*nr*1_ represents the first row of *M_new_* and $\vec{L_{s}}$ can be expressed as the Stokes vector: $\left[\begin{array}{l} I_{s}\\ Q_{s}\\ U_{s}\\ V_{s} \end{array}\right]$.

## 3. Experimental Results and Discussion

The proposed 6SV–SNR coupling model was used to simulate the atmospheric radiological transmission process of the Science instrument (coordinates: E116.3, N39.9) and to estimate the SNR of the remote sensor on 27 February 2022, from 11:00–11:30.

**Figure 3:**
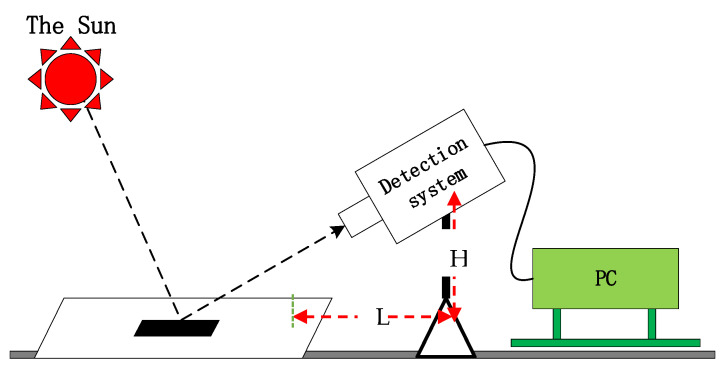
Simulation diagram of the experimental process. In the experiment, natural light was used as the light source input in an open outdoor area with no shelter. Images under different detection conditions were collected by PC and images were collected in a short time. L is the horizontal distance between the detection system and the target, H is the vertical distance between the detection system and the target, and both are about 1.5 m.

As shown in Figure 3, the experiment was carried out in an open outdoor area with no shelter in sunny weather. Aluminum plates were selected as the detection target, and a tripod was used to control the zenith angle observed by the remote sensor to ensure that the detection target was located at the center of the remote sensor’s field of view. Furthermore, the detection conditions recorded during the experiment were used as the input of the 6SV–SNR coupling model to calculate the theoretical SNR. Some of the input parameters are shown in Table 1.

**Figure 4:**
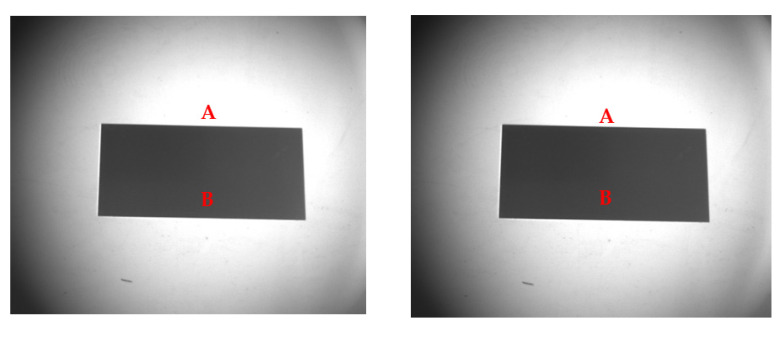
Image data at 532 nm visible band. (**Left**): the extinction ratio of the polarizer is 1000:1. (**Right**): the extinction ratio of the polarizer is 500:1. A is the background, B is the detection target (aluminum plates).

Through the remote sensor, several original images (50) were collected at four different permeation directions of 0°, 45°, 90°, and 135°, which correspond to the four visible wavelengths of 441 nm, 488 nm, 532 nm, and 610 nm, respectively. Figure 4 shows the image data of 90° transmissibility collected by the remote sensor in a visible light band of 532 nm. (Note: image data at 532 nm visible band are selected as representative).

The average gray value of each pixel is taken as the signal response value $\vec{S}(j,\lambda )$ of the remote sensor, and the root mean square of each pixel is taken as the total noise N(j,λ). The actual SNR of the remote sensor is then calculated. The theoretical SNR of the remote sensor under the same condition was obtained by substituting the detection condition parameters into the 6SV–SNR coupling model. The image data obtained when the transmission polarization direction of the polarizer is 90° were selected as representative, and the SNR and relative error obtained by data processing are shown in Table 2.

As can be observed from the data in the above table, when the extinction ratio of the polarizer is 1000:1, the maximum relative error of each band is 5.03% and the minimum is 1.29%. When the extinction ratio of the polarizer is 500:1, the maximum relative error of each band is 3.65% and the minimum is 1.20%. Among the selected four bands, when the bands are small, the larger the extinction ratio and the larger the SNR; when the bands are large, the larger the extinction ratio and the smaller the SNR. In addition, the SNR of the remote sensor increases with the increase of the central wavelength of the detection spectrum.

We also calculated the relative errors of the actual and theoretical SNR under the condition of considering the extinction ratio of the polarizer and not considering the extinction ratio of the polarizer, respectively, and selected the polarizer with the extinction ratio of 1000:1 for the experiment. The data are shown in Table 3. Except for the visible band at 532 nm, the relative errors of the actual and theoretical SNR of the remote sensor are smaller when the extinction ratio of the polarizer is taken into account, which proves the correctness and feasibility of the model proposed in this paper.

**Figure 5:**
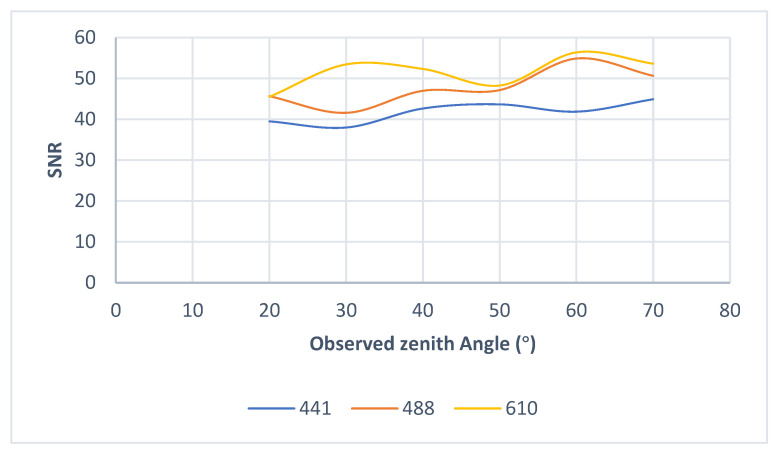
SNR curve with observed zenith angle. Three bands of 441 nm, 488 nm and 610 nm were selected to plot the change curve of SNR with observed zenith angle.

We also changed the observation zenith angle of the remote sensor under the same detection conditions to collect original images in four different transmission directions in the visible light bands of 441 nm, 488 nm, and 610 nm. Figure 5 shows the variation trend of the SNR of the remote sensor with the observed zenith angle when the extinction ratio of the polarizer is 1000:1 and the transmission direction is 90°.

It can be observed from the figure above that the SNR of the remote sensor is affected by the observation zenith angle. The longer the central wavelength of the detection spectrum, the higher the SNR of the remote sensor.

Through the above three sets of comparative experiments, it can be clearly seen that the theoretical SNR and its variation trend calculated by the 6SV–SNR coupling model proposed in this paper are consistent with the actual experimental results. In addition, the relative error between the theoretical value and the actual value was kept within a small range, which fully shows the validity of this model and its very important research significance in application. The SNR can be calculated more accurately through the model when a relatively accurate measurement of the actual detection conditions during detection is obtained, while it may be a shortage or constraint of the model.

## 4. Conclusions

In this study, the principle of polarization detection and noise detection are analyzed, and the SNR-estimation and testing method of the traditional imaging remote sensor is investigated. Based on the existing SNR model of the traditional imaging remote sensor, the internal SNR model of the polarization multispectral imaging remote sensor is established by considering the influence of the extinction ratio of the polarizer on polarization information; based on the study of atmospheric vector radiative transfer process, the 6SV–SNR coupling model is established by considering the influence of the detection environment. The new result is that the detection environment and extinction ratio all affect the SNR of the remote sensor, and the SNR increases with the increase in the central wavelength of the detection spectrum.

The proposed model is suitable for estimating the SNR of a typical polarization multispectral imaging remote sensor under different detection conditions, which solves the imperfect problem of the existing SNR-estimation methods for such remote sensors and provides an important basis for their application evaluation, especially in the selection of the detection environment and key components when using such remote sensors for detection. The next study has to be focused on the influence of the main direction error of the polarizer, the mechanical vibration of the remote sensor and other factors on the SNR.

## Figures and Tables

**Table 1:** The input parameters (the detection conditions recorded).

Solar Zenith Angle	Solar Azimuth	Probe Zenith Angle	Detect Azimuth	Atmospheric Model	Aerosol Type	Aerosol Concentration (Visibility)
38°	24°	50°	268°	Mid-latitude winter atmospheric model	City type	10 KM

**Table 2:** The SNR and relative error of each wavelength (different extinction ratios of polarizers).

	Extinction Ratio	Wavelength			
		441 nm	488 nm	532 nm	610 nm
**Actual value**	**1000:1**	43.6404	47.1396	45.0559	48.2649
	**500:1**	42.572	43.8246	49.1195	49.2864
**Theoretical value**	**1000:1**	43.078	45.3189	47.3208	50.6757
	**500:1**	43.0832	45.3246	47.3268	50.6821
**Relative error**	**1000:1**	1.29%	3.86%	5.03%	4.99%
	**500:1**	1.20%	3.42%	3.65%	2.83%

**Table 3:** The SNR and relative error of each wavelength (with or without extinction ratio).

	Consider Extinction Ratio?	Wavelength			
		441 nm	488 nm	532 nm	610 nm
**Actual value**		43.6404	47.1396	45.0559	48.2649
**Theoretical value**	**√ (yes)**	43.078	45.3189	47.3208	50.6757
	**× (no)**	43.0724	45.3132	47.3149	50.6694
**Relative error**	**√**	1.29%	3.86%	5.03%	4.99%
	**×**	1.30%	3.87%	5.01%	4.98%
